# Four New Briarane Diterpenoids from Taiwanese Gorgonian *Junceella fragilis*

**DOI:** 10.3390/md11062042

**Published:** 2013-06-10

**Authors:** Chia-Ching Liaw, Yu-Chi Lin, Yun-Sheng Lin, Chung-Hsiung Chen, Tsong-Long Hwang, Ya-Ching Shen

**Affiliations:** 1 School of Pharmacy, College of Medicine, National Taiwan University, Taipei 100, Taiwan; E-Mails: biogodas@hotmail.com (C.-C.L.); z10108042@email.ncku.edu.tw (Y.-C.L.); x00010106@meiho.edu.tw (Y.-S.L.); sionchen@ccms.ntu.edu.tw (C.-H.C.); 2 Department of Marine Biotechnology and Resources, National Sun Yat-Sen University, Kaohsiung 804, Taiwan; 3 Department of Life Sciences, National Cheng Kung University, No. 1 University Road, Tainan 701, Taiwan; 4 Graduate Institute of Natural Products, Chang Gung University, Taoyuan 333, Taiwan; E-Mail: htl@mail.cgu.edu.tw

**Keywords:** *Junceella fragilis*, briarane-type diterpenoids, frajunolides, anti-inflammatory

## Abstract

Four new 8-hydroxybriarane diterpenoids, frajunolides P–S (**1**–**4**), together with umbraculolide A, juncenolide C, junceellonoid A and juncin R, were isolated from the acetone extract of the gorgonian *Junceella fragilis*, collected from the southeast coast of Taiwan. Compound **1** contains an unusual pivaloyloxy group at C-2, while **3** is a rare compound having a chlorine atom on the olefinic carbon (C-6). The structures of the isolated compounds were established by extensive spectroscopic analysis, including 1D- and 2D-NMR, as well as HRMS data. Compound **1** was further confirmed by X-ray crystallographic analysis. In the anti-inflammatory test, compounds **1** and **2** exhibited moderate inhibition on superoxide anion generation and elastase release by human neutrophils in response to formylmethionylleucyl-phenylalanine/dihydrocytochalasin B (fMLP/CB).

## 1. Introduction

**Figure 1:**
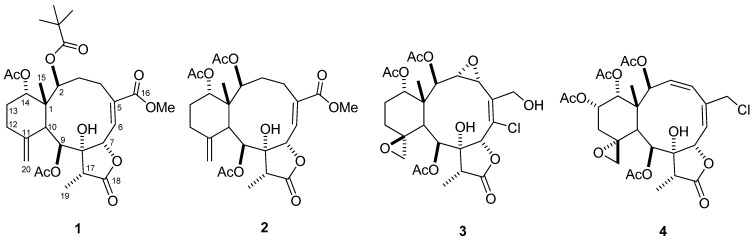
Frajunolides P–S (**1**–**4**) isolated from gorgonian *J. fragilis*.

Marine invertebrates, especially gorgonian octocorals, have been proven to be rich and important sources of natural products as lead compounds in drug discovery. Members of the gorgonians, *Junceella* and *Briareum*, have yielded numerous and highly oxygenated briarane-type diterpenes with a γ-lactone ring, produced from 3,8-cyclized cembranoids [1,2,3]. Many of the briarane diterpenoids have been reported to exhibit interesting biological activities, such as cytotoxic [4,5,6], anti-inflammatory [7,8], antiviral [8], insecticidal [9] and immunomodulatory [10] activities. Our previous chemical investigation of the genus *Junceella* has resulted in the isolation of over 20 briaranes, including frajunolides A–O and juncenolides A–O [11,12,13,14,15,16,17,18]. As part of our continuing search for bioactive natural products, the chemical constituents from other chromatographic fractions of *J. fragilis* were investigated. Herein, we report the isolation and structural elucidation of four additional new 8-hydroxybriarane diterpenoids, frajunolides P–S (Figure 1, **1**–**4**), from the acetone extract of this source, collected from the southeast coast of Taiwan. Their anti-inflammatory activities were tested and evaluated by superoxide anion generation and elastase release by human neutrophils in response to formylmethionylleucyl-phenylalanine/dihydrocytochalasin B (fMLP/CB).

## 2. Results and Discussion

**Figure 2:**
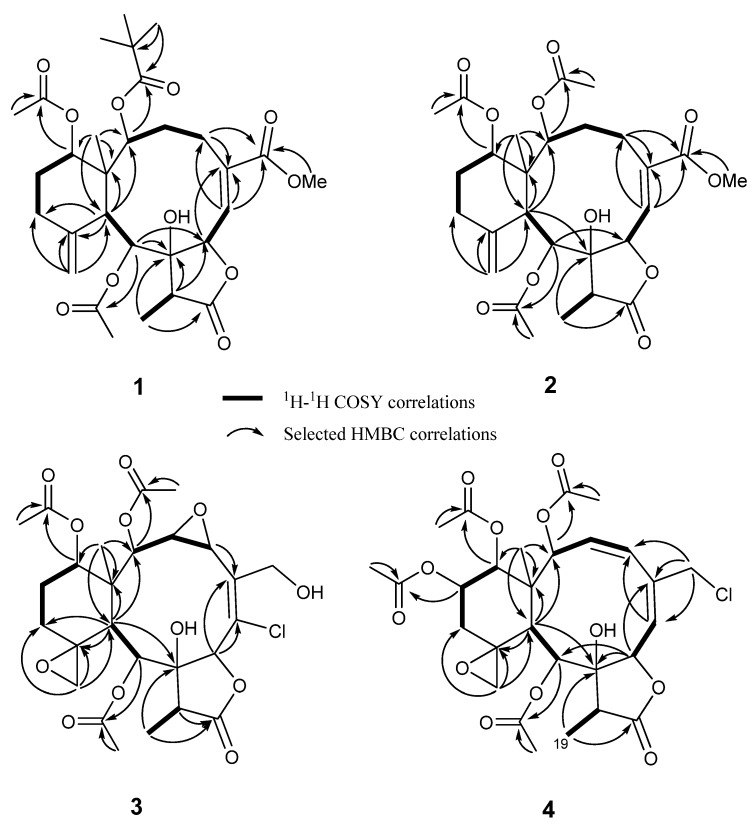
^1^H–^1^H COSY and HMBC correlations of compounds **1**–**4**.

Compound **1**, [α]_D_^25^ +4 (*c* 0.5 CH_2_Cl_2_), was isolated as colorless prisms and had a molecular formula of C_30_H_42_O_11_ deduced from HRESIMS (*m/z* 601.2620 [M + Na]^+^, calcd. for C_30_H_42_O_11_Na, 601.2625), indicating ten degrees of unsaturation. The IR spectrum of compound **1** exhibited diagnostic absorption bands of hydroxyl (3443 cm^−1^), γ-lactone (1776 cm^−1^), ester carbonyl (1722 cm^−1^) and conjugated ketone (1655 cm^−1^) functionalities. The ^1^H and ^13^C NMR spectroscopic data (Table 1, Table 2) indicated the presence of a methyl singlet (δ_H_ 1.08; δ_C_ 16.5, C-15), a methyl doublet (δ_H_ 1.19, *J* = 7.0 Hz; δ_C_ 8.5, C-19), one exocyclic double bond (δ_H_ 5.02, 4.98, each s, H_2_-20; δ_C_ 112.5, C-20; δ_C_ 149.7, C-11), one trisubstituted double bond (δ_H_ 6.89, d, *J* = 9.6 Hz, H-6; δ_C_ 134.5, C-5; 136.8, C-6), four oxygenated methine protons and carbons, (δ_H_ 5.11, d, *J* = 7.6 Hz; δ_C_ 75.6, C-2; δ_H_ 5.31, d, *J* = 9.6 Hz; δ_C_ 78.3, C-7; δ_H_ 5.60, d, *J* = 2.8 Hz; δ_C_ 72.8, C-9; δ_H_ 4.64, t, *J* = 2.8 Hz; δ_C_ 74.1, C-14), an oxygenated quaternary carbon (δ_C_ 83.5, C-8), four methylene carbons (δ_C_ 32.3, 24.1, 31.4, 28.9) and two methine carbons (δ_C_ 43.9 and 44.7), together with a conjugated ester carbonyl (δ_C_ 166.8, C-16) and γ-lactone carbonyl carbon (δ_C_ 174.4, C-19). Detailed analysis of spectroscopic data of **1** and comparison with the related structures of the genus *Junceella* suggested that compound **1** is a highly oxygenated briarane-type diterpenoid with a fused γ-lactone ring similar to juncenolide O, previously isolated from *J. juncea* [18]. In addition, the remaining NMR spectroscopic data contained a methoxy group (δ_H_ 3.81), two acetate groups (δ_H_ 2.20, 1.92, each 3H) and a pivaloyloxy group (δ_H_ 1.38 × 3, 9H). Furthermore, the HMBC correlation showed that the latter was located at C-2, while the acetyl groups were located at C-9 and C-14, and the methoxy group was attached at C-16. The complete planar structure of **1** was further confirmed by the ^1^H–^1^H COSY and HMBC correlations (Figure 2).

**Table 1:** ^1^H-NMR spectroscopic data for compounds **1**–**4**. (δ in ppm, *J* in Hz).

No.	1	2	3	4
2	5.11 (d, *J* = 7.6)	5.00 (m)	4.83 (d, *J* = 9.2)	5.33 (d, *J* = 9.2)
3	2.53 (m)	2.48 (m)	3.40 (dd, *J* = 9.6, 3.6)	5.57 (dd, *J* = 10.4, 9.6)
	1.80 (m)	1.81 (m)		
4	2.52 (m)	2.81 (m)	4.08 (d, *J* = 3.6)	6.30 (d, *J* = 10.4)
	2.74 (m)	2.51 (m)		
6	6.89 (d, *J* = 9.6)	6.84 (d, *J* = 10.0)	-	5.96 (d, *J* = 8.8)
7	5.31 (d, *J* = 9.6)	5.32 (d, *J* = 10.0)	5.41 (s)	4.91 (d, *J* = 8.8)
9	5.60 (d, *J* = 2.8)	5.56 (d, *J* = 3.5)	5.64 (d, *J* = 8.0)	4.68 (d, *J* = 5.2)
10	3.38 (d, *J* = 2.8)	3.27 (d,* J* = 3.5)	2.49 (d, *J* = 8.0)	3.02 (d, *J* = 5.2)
12	2.23 (m)	2.25 (m, 2H)	2.18 (m)	2.47 (td, *J* = 12.4, 1.6)
	1.80 (m)	-	1.78 (m)	1.32 (dd, *J* = 13.2, 3.6)
13	1.80 (m)	1.81 (m, 2H)	2.30 (m)	4.95 (ddd, *J* = 12.8, 4.0, 2.8)
	1.40 (m)		1.12 (m)	
14	4.64 (t, *J* = 2.8)	4.71 (t, *J* = 3.5)	4.88 (d, *J* = 5.2)	5.18 (br s)
15	1.08 (s)	1.25 (s)	1.24 (s)	1.09 (s)
16	-	-	4.57 (dd,* J* = 12.4, 8.8)	4.56 (s, 2H)
	-	-	4.31 (dd,* J* = 12.4, 6.0)	-
17	2.63 (q, *J* = 7.2)	2.60 (q, *J* = 7.0)	2.26 (q, *J* = 7.2)	2.26 (q, *J* = 6.8)
19	1.19 (d, *J* = 7.2)	1.19 (d, *J* = 7.0)	1.22 (d, *J* = 7.2)	1.12 (d, *J* = 6.8)
20	5.02 (s)	5.04 (s)	2.98 (d, *J* = 4.4)	3.52 (br s)
	4.98 (s)	4.99 (s)	2.80 (d, *J* = 4.0)	2.72 (d, *J* = 2.4)
2-OCOCH_3_	1.92 (s)	1.97 (s)	2.10 (s)	1.93 (s)
9-OCOCH_3_	2.20 (s)	2.23 (s)	2.24 (s)	2.16 (s)
13-OCOCH_3_	-	-	-	2.07 (s)
14-OCOCH_3_	-	1.93 (s)	1.96 (s)	1.95 (s)
2-OCOC(CH_3_)_3_	1.38 (s, 9H)	-	-	-
16-OCH_3_	3.81 (s)	3.82 (s)	-	-
8-OH	-	-	5.86 br s	-
16-OH	-	-	3.72 (dd, *J* = 8.0, 6.0)	-

**Table 2:** ^13^C-NMR spectroscopic data for compounds **1**–**4** (δ in ppm, mult).

No.	1	2	3	4
1	48.8 (s)	47.4 (s)	44.7 (s)	46.4 (s)
2	75.6(d)	73.6 (d)	75.9 (d)	74.1 (d)
3	32.3 (tH)	30.9 (t)	58.7 (d)	131.7 (d)
4	24.1 (t)	22.8 (t)	59.0 (d)	128.0 (d)
5	134.5 (s)	134.2 (s)	135.7 (s)	139.9 (s)
6	136.8 (d)	138.1 (d)	132.4 (d)	126.0 (d)
7	78.3 (d)	77.5 (d)	75.7 (d)	78.4 (d)
8	83.5 (s)	83.5 (s)	81.2 (s)	80.8 (s)
9	72.8 (d)	72.8 (d)	66.8 (d)	64.2 (d)
10	43.9 (d)	43.2 (d)	40.2 (d)	37.4 (d)
11	149.7 (s)	150.5 (s)	61.2 (s)	58.1 (s)
12	31.4 (t)	29.4 (t)	24.1 (t)	34.2 (t)
13	28.9 (t)	27.3 (t)	24.3 (t)	67.6 (d)
14	74.1 (d)	74.8 (d)	72.7 (d)	73.7 (d)
15	16.5 (q)	15.0 (q)	14.7 (q)	14.3 (q)
16	166.8 (s)	168.0 (s)	58.8 (s)	44.6 (s)
17	44.7 (d)	42.9 (d)	42.9 (d)	43.8 (d)
18	174.4 (s)	175.4 (s)	174.8 (s)	175.2 (s)
19	8.5 (q)	6.6 (q)	6.4 (q)	6.3 (q)
20	112.5 (d)	112.7 (d)	58.4 (t)	50.1 (t)
2-OCOCH_3_	-	170.0 (s)	171.2 (s)	170.0 (s)
2-OCOCH_3_	-	20.9 (q)	20.9 (q)	20.8 (q)
9-OCOCH_3_	168.3 (s)	169.3 (s)	169.2 (s)	170.2 (s)
9-OCOCH_3_	23.3 (q)	21.7 (q)	21.9 (q)	21.5 (q)
13-OCOCH_3_	-	-	-	170.2 (s)
13-OCOCH_3_	-	-	-	21.0 (q)
14-OCOCH_3_	169.6 (s)	170.5 (s)	170.2 (s)	170.0 (s)
14-OCOCH_3_	23.0 (q)	21.2 (q)	21.0 (q)	21.3 (q)
2-OCOC(CH_3_)_3_	174.7 (s)	-	-	-
2-OCOC(CH_3_)_3_	- ^a^	-	-	-
2-OCOC(CH_3_)_3_	28.0 (q)	-	-	-
16-OCH_3_	53.6 (q)	52.5 (q)	-	-

^a^ Signal not observed.

**Figure 3:**
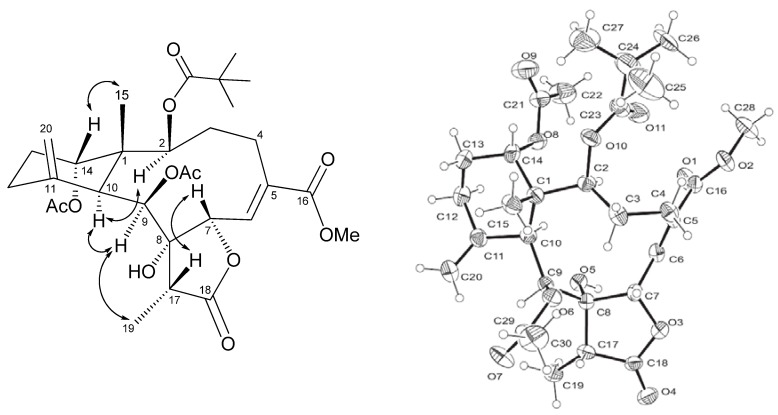
Key NOESY correlations and X-ray crystallographic diagram of compound **1**.

The configuration of the Me-15 in naturally occurring briaranes was previously assigned β-orientation, and H-10 was in the α-orientation. In the NOESY spectrum of **1** (Figure 3), correlations of Me-15/H-14, H-10/H-2, H-10/H-9, H-9/Me-19 and H-7/H-17 suggested that H-7, Me-15 and H-17 were all β-oriented, while H-2, H-9 and H-14 were α-disposition. Finally, the absolute configuration of compound **1** was unambiguously established by a single-crystal X-ray diffraction, as illustrated in Figure 3. Hence, compound **1** was determined as (1*S*,2*S*,6*Z*,7*S*,8*R*,9*S*,10*S*,14*S*,17*R*)-2-pivaloyloxy-9,14-diacetoxy-8-hydroxybriaran-5(6)*Z*-dien-18,7-olide, and the name frajunolide P was given.

Compound **2** was isolated as a colorless amorphous gum and had the molecular formula C_27_H_36_O_11_, as determined by HRESIMS and distortionless enhancement by polarization transfer (DEPT) NMR analysis. The presence of a hydroxyl, an ester group and a γ-lactone were consistent with IR absorption bands at 3443, 1736 and 1780 cm^−1^, respectively. It was found that the ^1^H- and ^13^C NMR spectroscopic data (Table 1, Table 2) were similar to those of compound **1**, except that the pivaloyloxy group at C-2 was replaced by an acetate group (δ_H_ 1.97; δ_C_ 170.0, 20.9). This was confirmed by the HMBC correlation (Figure 2) between H-2 (δ_H_ 5.00) and the carbonyl carbon at δ_C_ 170.0. The planar structure and NMR assignments for **2** were established by detailed analysis of 2D NMR, including ^1^H–^1^H COSY, HMQC and HMBC correlations. The configurations of compound **2** were determined by observation of NOESY correlations and on the basis of biogenetic consideration similar to compound **1**. Therefore, compound **2** was identified as (1*S*,2*S*,6*Z*,7*S*,8*R*,9*S*,10*S*,14*S*,17*R*)-2,9,14-triacetoxy-8-hydroxybriaran-5(6)-dien-18,7-olide and named frajunolide Q.

**Figure 4:**
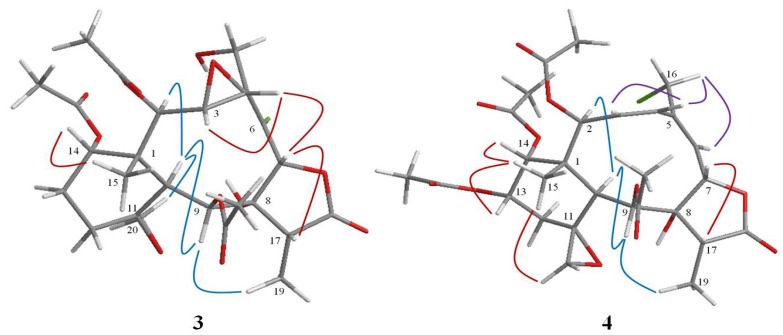
Key NOESY correlations and computer-generated perspective model of compounds **3** and **4**.

The molecular formula, C_26_H_33_O_12_Cl, of compound **3** was obtained from the HRESIMS, which showed a *quasi*-molecular ion peak at *m/z* 595.1559 [M + Na]^+^. The presence of a chlorine atom was suggested from an isotope ion at *m/z* 597.1536 [M + Na]^+^, which exhibited one-third of the relative intensity of the normal ion peak. The IR spectrum showed absorption bands at 3480, 3241, 1782 and 1742 cm^−1^, indicating the presence of two hydroxyl, γ-lactone and ester carbonyl functionalities. The ^1^H and ^13^C NMR spectroscopic data (Table 1, Table 2) further supported the existence of three acetate groups (δ_C_ 171.2, 170.2, 169.2, 21.9, 21.0, 20.9), assigned to C-2 (δ_C_ 75.9), C-9 (δ_C_ 66.8) and C-14 (δ_C_ 72.7) with the aid of HMBC correlations between H-2 (δ_H_ 4.83, d, *J* = 9.2 Hz), H-9 (δ_H_ 5.64, d, *J* = 8.0 Hz), H-14 (δ_H_ 4.88, d, *J* = 5.2 Hz) and acetate carbonyls, respectively. The remaining ^1^H and ^13^C NMR signals revealed that compound **3** possessed a methyl singlet (δ_H_ 1.24, Me-15), a methyl doublet (δ_H_ 1.22, d, *J* = 7.2 Hz, Me-19), one epoxy ring (δ_C_ 58.7, 59.0; δ_H_ 3.40, dd, *J* = 9.6, 3.6 Hz; 4.08, d, *J* = 3.6 Hz), one spirocyclic oxirane ring (δ_C_ 61.2, 58.4; δ_H_ 2.98, d, *J* = 4.4 Hz), 2.80, d, *J* = 4.0 Hz), one tetrasubstituted double bond (δ_C_ 135.7, 132.4), two methylene carbons (δ_C_ 24.3, 24.1), one oxymethylene (δ_C_ 58.8; δ_H_ 4.57, dd, *J* = 12.4, 8.8 Hz; 4.31, dd, *J* = 12.4, 6.0 Hz), one oxymethine (δ_C_ 75.7; δ_H_ 5.41, s), two methine protons (δ_H_ 2.49, d, *J* = 8.0 Hz; 2.26, q, *J* = 7.2 Hz) and two quaternary carbons (δ_C_ 44.7,C-1; 81.2, C-8), together with γ-lactone carbonyl carbon at δ_C_ 174.4 (C-18). The above observation agreed with a 8-hydroxybriarane with γ-lactone in **3**. The structure was further established by detailed analysis of 2D NMR. The signals of the H-20 showed HMBC correlations (Figure 2) with C-11, C-12 and C-10, indicating an epoxy ring at C-11 (δ_C_ 61.2)/C-20 (δ_C_ 58.4). The other epoxy ring was located at C-3/C-4 by observation of COSY (H-2/H-3/H-4) and HMBC correlations between H-4 and C-5. Finally, the chlorine atom has to be attached to C-6 (δ_C_ 132.4) of the tetrasubstituted double bond. This was confirmed by comparison with the NMR data of briarein F [19]. The configuration of compound **3** (Figure 4) was determined by a NOESY experiment and coupled with molecular model MM2 minimized energy calculation [20]. The NOESY spectrum showed correlations of H-3/H-4, H-7/H-4, H-17 and Me-15/H-14 indicated that H-3, H-4, H-7, H-14, H-17 and Me-15 are all β-orientation, while H-2, H-9 and Me-19 favored α disposition, due to correlations of H-10/H-2, H-9 and H-9/Me-19. Moreover, the configuration at C-11 was assigned as *S*, which was determined by the NOESY correlation of H-10/H-20 and comparison with ^13^C NMR data of the related literature [21]. The above interpretation suggested that compound **3** was a novel 8-hydroxybriarane possessing a chlorine atom at C-6, and thus, the name frajunolide R was given.

The HRESIMS of **4** exhibited two *pseudo*-molecular ion peaks at *m/z* 621.1716 [M + Na]^+^ and 623.1690 [M + Na + 2]^+^, accounting for a chlorine atom in the molecular formula, C_28_H_35_O_12_Cl. The IR spectrum showed absorption bands of a hydroxyl (3467 cm^−1^), a γ-lactone (1780 cm^−1^) and an ester carbonyl (1739 cm^−1^) group. The ^1^H- and ^13^C-NMR spectroscopic data (Table 1, Table 2) of **4** resembled those of juncenolide B, previously isolated from *J. juncea* [15], suggesting that they were analogs. Detailed analysis of NMR and MS data concluded that the only difference between them was the presence of a chlorine atom at C-16 in **4**, replacing the original hydroxyl group in juncenolide B. This finding was supported by observation of the chemical shift of C-6 at δ_C_ 44.6. The relative configuration of **4** was determined by comparing the proton coupling constants of **4** with those of juncenolide B and NOESY studies. Molecular modeling based on MM2 minimized energy was calculated to confirm the structure as illustrated in Figure 4. Thus, compound **4** was elucidated as a 16-chlorinated derivative of juncenolide B, and the name frajunolide S was given.

Four known briaranes were also isolated and identified as umbraculolide A [22], juncenolide C [15], junceellonoid A [23] and juncin R [24], respectively, by comparison with the spectroscopic data reported in the literature. The anti-inflammatory activities (Table 3) of briaranes **1**–**4** were tested and evaluated for their inhibition of elastase release and generation of superoxide anion by human neutrophils in response to fMet-Leu-Phe (fMLP)/cytochalasin B. Compounds **1** and **2** showed moderate inhibitory activities on both superoxide anion generation and elastase release at 10 μg/mL.

**Table 3:** Effects of compounds on superoxide anion generation and elastase release by human neutrophils in response to formylmethionylleucyl-phenylalanine/dihydrocytochalasin B (fMLP/CB).

Compound	Superoxide anion	Elastase release
	Inhibition (%)	Inhibition (%)
**1**	32.5 ± 1.5 ***	35.6 ± 3.2 *
**2**	28.7 ± 3.4 *	34.1 ± 2.9 **
**3**	9.70 ± 1.3 **	16.0 ± 5.3 *
**4**	5.80 ± 3.0	−4.5 ± 3.4

Percentage of inhibition (%) at 10 μg/mL concentration. Results are presented as the mean ± S.E.M. (*n* = 3). * *p* < 0.05, ** *p* < 0.01, *** *p* < 0.001 compared with the control value.

## 3. Experimental Section

### 3.1. General Experimental Procedures

Optical rotations were recorded on a JASCO DIP-1000 polarimeter. IR spectra were measured on Hitachi T-2001 spectrophotometer. LRESIMS and HRESIMS were taken on a JEOL JMS-HX 110 mass spectrometer. The ^1^H, ^13^C NMR, ^1^H–^1^H COSY, HMQC, HMBC and NOESY spectra were recorded on a Varian MR 400 and UNITY INOVA 500 spectrometers. The chemical shifts were given in δ (ppm) and coupling constants in Hz. Silica gel 60 (Merck) was used for column chromatography, and pre-coated silica gel plates (Merck, Kieselgel 60 F-254, 1 mm) were used for preparative TLC. Sephadex LH-20 (Amersham Pharmacia Biotech AB, Sweden) was used for separation. LiChrospher^®^ Si 60 (5 μm, 250-10, Merck, Germany) and LiChrospher^®^ 100 RP-18e (5 μm, 250-10, Merck, Germany) were used for NP-HPLC and RP-HPLC (Hitachi), respectively.

### 3.2. Animal Material

The gorgonian *Junceella fragilis* Ridley (Ellisellidae) was collected in Tai-Tong County, Taiwan, by scuba diving at a depth of 15 m, in February 2006. The fresh gorgonian was immediately frozen after collection and kept at −20 °C until processed. A voucher specimen (WSG-5) was deposited in the School of Pharmacy, College of Medicine, National Taiwan University, Taiwan.

### 3.3. Extraction and Isolation

The gorgonian *J. fragilis* (wet, 3.9 kg) was minced and extracted with acetone (3 × 5 L) at room temperature, and the acetone extract was concentrated under vacuum. The crude extract (33 g) was partitioned between EtOAc and H_2_O (1:1). The EtOAc-soluble portion (24 g) was shaken with *n*-hexane-MeOH–H_2_O (4:3:1), and the MeOH layer was evaporated and separated on Sephadex LH-20 to give eight fractions (L1 to L8). Fraction L3 (3 g) was subjected to column chromatography using silica gel and a gradient of *n*-hexane/CH_2_Cl_2_/MeOH to obtain 33 fractions (L3-1 to L3-33). Fraction L3-14 (111 mg) was separated on NP-HPLC using *n*-hexane/CH_2_Cl_2_/MeOH (40:20:1) to yield **1** (3.5 mg) and **2** (1.0 mg). Fraction L3-17 (104 mg) was subjected to RP-HPLC using MeOH/H_2_O/CH_3_CN (70:25:5) to give **4** (7.5 mg), umbraculolide A (18 mg) and junceellonoid A (16 mg). L3-20 (97 mg) was separated on RP HPLC using MeOH/H_2_O/CH_3_CN (70:25:5) to obtain **3** (3.5 mg), junceellolide C (12 mg) and juncin R (2.8 mg).

Frajunolide P (**1**): colorless prisms; [α]_D_^24^ +4 (*c* 0.5, CH_2_Cl_2_); IR ν_max _3443, 2934, 1776, 1722, 1655, 1379, 1267, 1220 cm^−1^; ^1^H NMR data (400 MHz, CDCl_3_), see Table 1; ^13^C NMR data (100 MHz, CDCl_3_), see Table 2; ESIMS *m/z* 601 [M + Na]^+^; HRESIMS *m/z* 601.2620 [M + Na]^+^ (calcd. for C_30_H_42_O_11_Na, 601.2625).

Frajunolide Q (**2**): colorless amorphous gum; [α]_D_^24^ +32 (*c* 0.1, CH_2_Cl_2_); IR ν_max_ 3443, 2923, 1780, 1736, 1645, 1375, 1264, 1219 cm^−1^; ^1^H NMR data (500 MHz, CDCl_3_), see Table 1; ^13^C NMR data (125 MHz, CDCl_3_), see Table 2; ESIMS *m/z* 559 [M + Na]^+^; HRESIMS *m/z* 559.2156 [M + Na]^+^ (calcd. for C_2__7_H_3__6_O_1__1_Na, 559.2155).

Frajunolide R (**3**): colorless amorphous gum; [α]_D_^24^ +13 (*c* 0.3, CH_2_Cl_2_); IR ν_max_ 3480, 3241, 2926, 2856, 1782, 1742, 1373, 1252, 1212 cm^−1^; ^1^H NMR data (400 MHz, CDCl_3_), see Table 1; ^13^C NMR data (100 MHz, CDCl_3_), see Table 2; ESIMS *m/z* 595 [M + Na]^+^, *m/z* 597 [M + Na + 2]^+^; HRESIMS *m/z* 595.1559 [M + Na]^+^ (calcd. for C_26_H_33_^35^ClO_12_Na, 595.1558).

Frajunolide S (**4**): colorless amorphous gum; [α]_D_^24^ −22.0 (*c* 0.2, CH_2_Cl_2_); IR ν_max_ 3476, 2947, 1780, 1739, 1372, 1250, 1223 cm^−1^; ^1^H NMR data (400 MHz, CDCl_3_), see Table 1; ^13^C NMR data (100 MHz, CDCl_3_), see Table 2; ESIMS *m/z* 621 [M + Na]^+^, *m/z* 623 [M + Na + 2]^+^; HRESIMS *m/z* 621.1716 [M + Na]^+^ (calcd. for C_28_H_35_^35^ClO_1__2_Na, 621.1715).

### 3.4. Single Crystal X-ray Structure Determination of Frajunolide P (**1**)

A suitable colorless crystal (0.37 × 0.14 × 0.08 mm^3^) of **1** for diffraction was obtained by simple evaporation from methanol solution. Crystal data: C_30_H_42_O_11_ orthorhombic, *a* = 10.1174(2) Å, *b* = 14.0223(3) Å, *c* =21.0529(5) Å, *V* = 2986.76(11) Å^3^, space group P22_1_2_1_, *Z* = 4, D_calcd_ 1.287 mg/m^3^, λ = 0.71073 Å, μ(Mo Kα) 90.098 mm^−1^, *F*(000) = 1240, *T* = 293(2) K. A total of 18,766 reflections collected, of, which 5275 unique reflections (*R*_int_ = 0.0770) with I > 2σ(*I*) were used for the analysis. The data was solved using the direct method, and the structure was refined by full-matrix least-squares procedure on *F*^2^ values. The refined structural model converged to a final *R*1 0.0684, *wR*2 0.1808 with goodness-of-fit = 1.036. The final X-ray molecular model is shown in Figure 3.

### 3.5. Anti-Inflammatory Assays

#### 3.5.1. Human Neutrophils Elastase Release

Degranulation of azurophilic granules was determined by elastase release, as described previously [25]. Experiments were performed using MeO-Suc-Ala-Ala-Pro-Val-*p*-nitroanilide as the elastase substrate. After supplementation with MeO-Suc-Ala-Ala-Pro-Val-*p*-nitroanilide (100 μM), neutrophils (6 × 10^5^ cell/mL) were equilibrated at 37 °C for 2 min and incubated with each test compound for 5 min. Cells were activated by fMLP (100 nM)/CB (0.5 μg/mL), and changes in absorbance at 405 nm were monitored continuously for elastase release. The results are expressed as the percentage of the initial rate of elastase release in the fMLP/CB-activated, test compound-free (DMSO) control system.

#### 3.5.2. Human Neutrophil Superoxide Generation

Human neutrophils were obtained by means of dextran sedimentation and Ficoll centrifugation. Superoxide anion production was assayed by monitoring the superoxide dismutase-inhibitable reduction of ferricytochrome *c*. In brief, after supplementation with 0.5 mg/mL ferricytochrome *c* and 1.0 mM Ca^2+^, neutrophils were equilibrated at 37 °C for 2 min and incubated with drugs for 5 min. Cells were activated with 100 nM fMLP for 10 min. When fMLP was used as a stimulant, CB (1 μg/mL) was incubated for 3 min before activation by the peptide (fMLP/CB). Changes in absorbance with the reduction of ferricytochrome *c* at 550 nm were continuously monitored in a double-beam, six-cell positioner spectrophotometer with constant stirring (Hitachi U-3010, Tokyo, Japan). Calculations were based on differences in the reactions with and without superoxide dismutase (SOD, 100 U/mL) divided by the extinction coefficient for the reduction of ferricytochrome *c*.

## 4. Conclusions

Our continuing investigation on constituents of Taiwanese gorgonian *Junceella fragilis* has resulted in the isolation of eight 8-hydroxybriarane diterpenoids, including four new ones, frajunolides P–S (**1**–**4**). In the anti-inflammatory effects on elastase release and generation of superoxide anion by human neutrophils, compounds **1** and **2** exhibited moderate inhibitory activities.

## Supplementary Files

Supplementary File 1 Supplementary Information (PDF, 1096 KB)
