# Supplementary material for: Four New Briarane Diterpenoids from Taiwanese Gorgonian Junceella fragilis
Source: Mar Drugs. 2013 Jun 10;11(6):2042–53. doi: 10.3390/md11062042 (PMC3721220; doi:10.3390/md11062042)

Supplementary Information

Figure S1. ^{1}H NMR spectrum (400 MHz, $CDCl_3$) of frajunolide P (**1**). 2

Figure S2. ^{13}C NMR spectrum (100 MHz, $CDCl_3$) of frajunolide P (**1**). 3

Figure S3. ^{1}H NMR spectrum (500 MHz, $CDCl_3$) of frajunolide Q (**2**). 4

Figure S4. ^{13}C NMR spectrum (125 MHz, $CDCl_3$) of frajunolide Q (**2**). 5

Figure S5. ^{1}H NMR spectrum (400 MHz, $CDCl_3$) of frajunolide R (**3**). 6

Figure S6. ^{13}C NMR spectrum (100 MHz, $CDCl_3$) of frajunolide R (**3**). 7

Figure S7. ^{1}H NMR spectrum (400 MHz, $CDCl_3$) of frajunolide S (**4**). 8

Figure S8. ^{13}C NMR spectrum (100 MHz, $CDCl_3$) of frajunolide S (**4**). 9

Figure S1. ^{1}H NMR spectrum (400 MHz, $CDCl_3$) of frajunolide P (**1**).

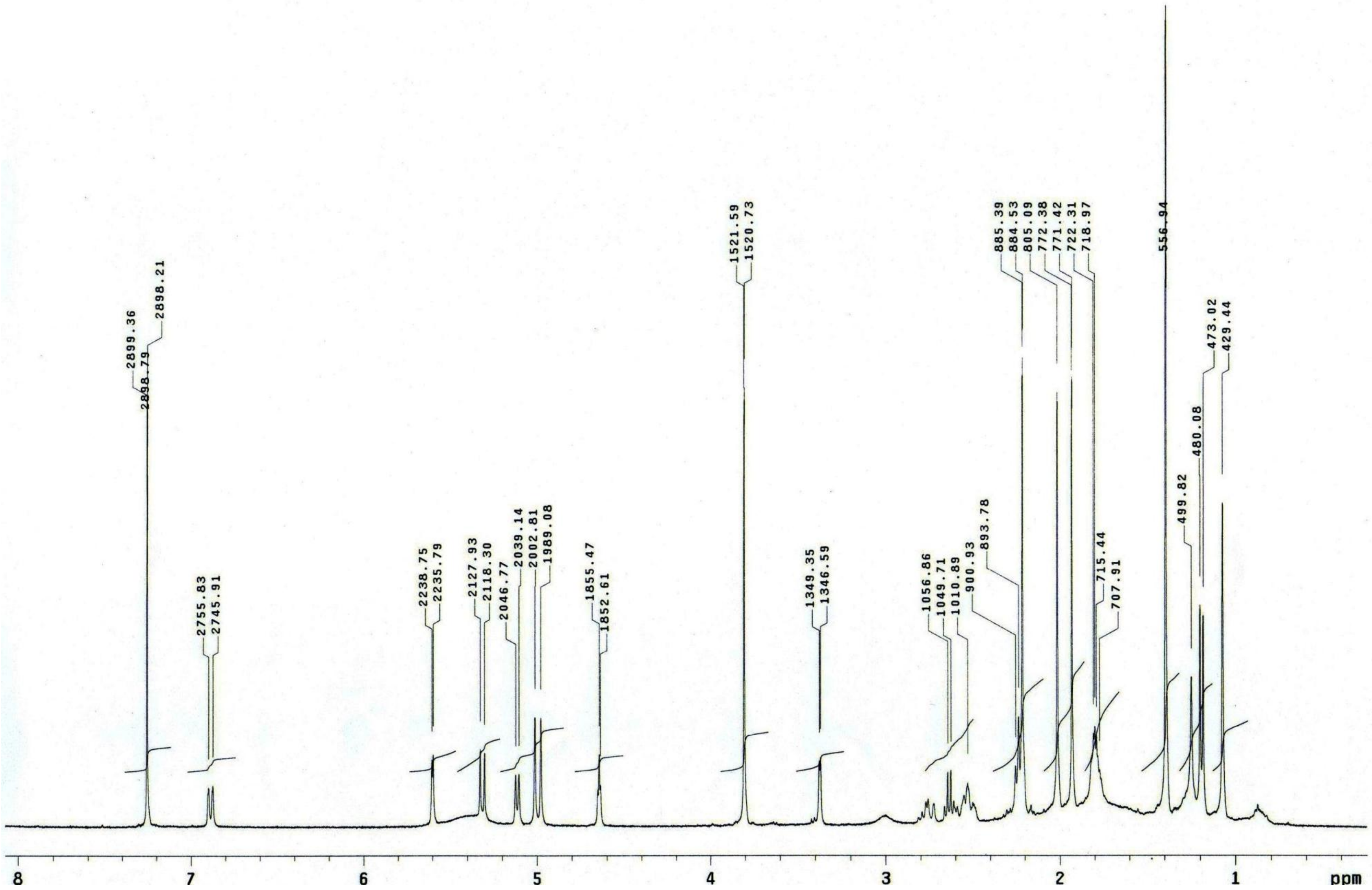

Figure S2. ^{13}C NMR spectrum (100 MHz, $CDCl_3$) of frajunolide P (**1**).

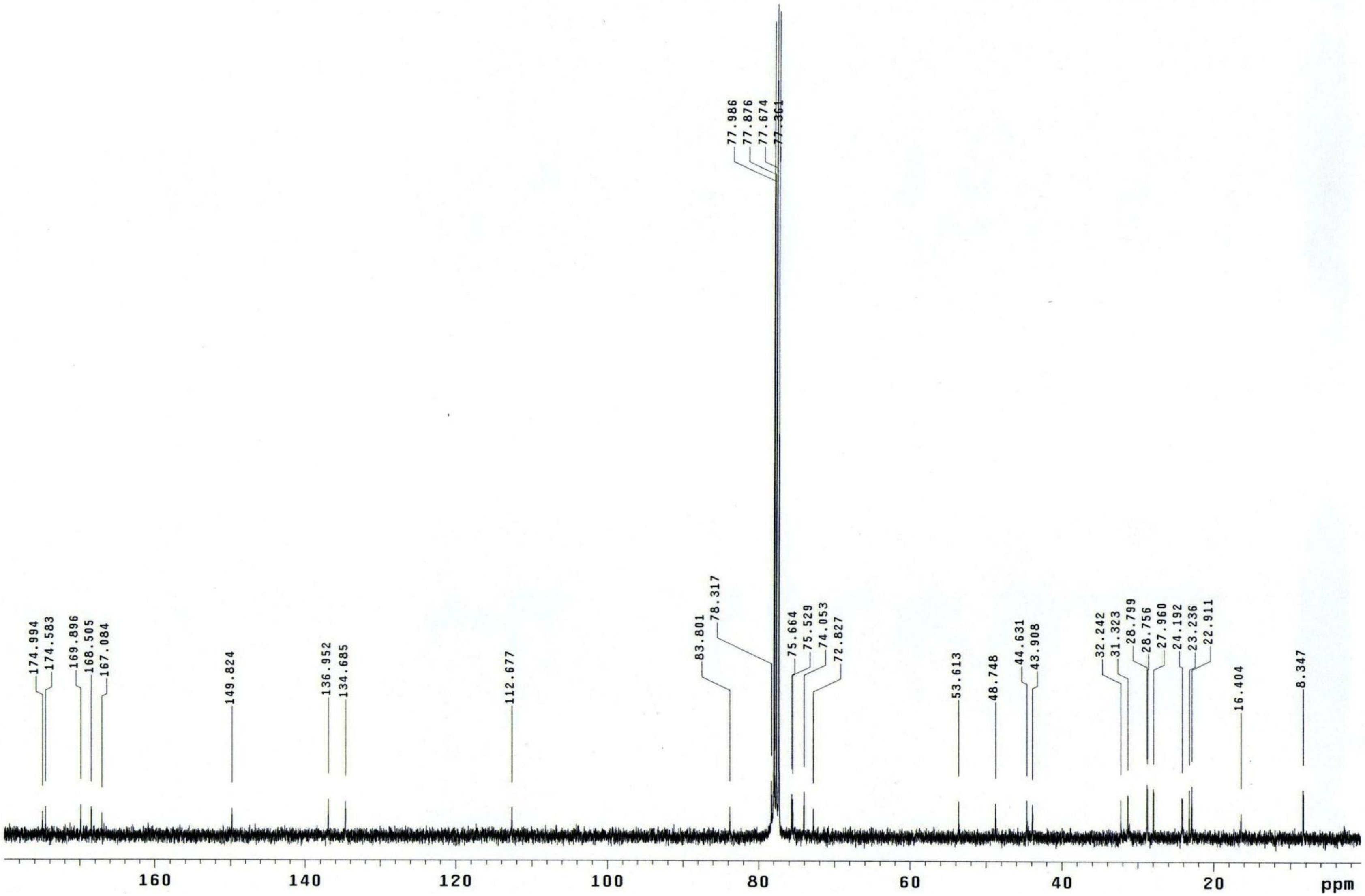

Figure S3. ^{1}H NMR spectrum (500 MHz, $CDCl_3$) of frajunolide Q (**2**).

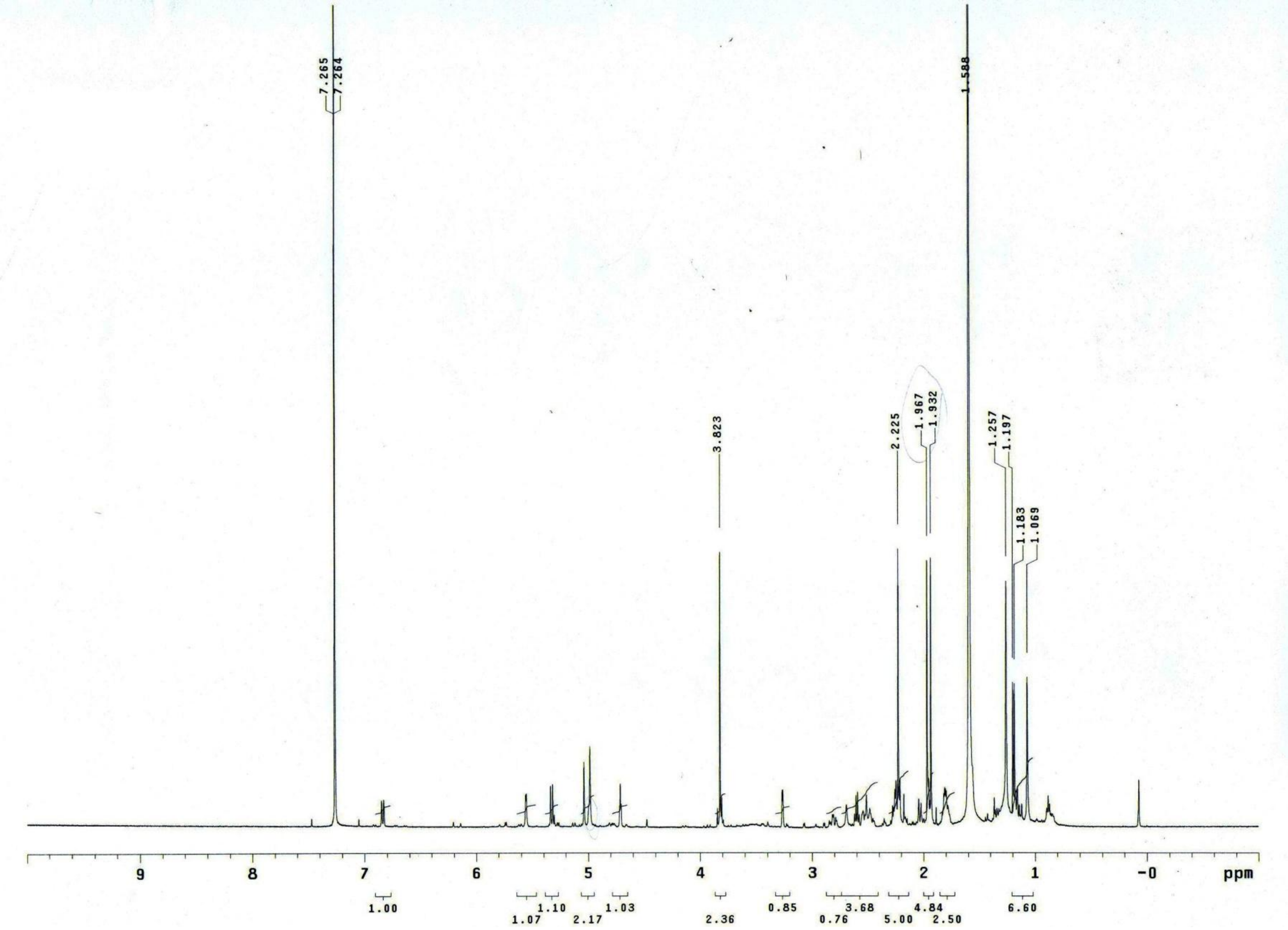

Figure S4. ^{13}C NMR spectrum (125 MHz, $CDCl_3$) of frajunolide Q (**2**).

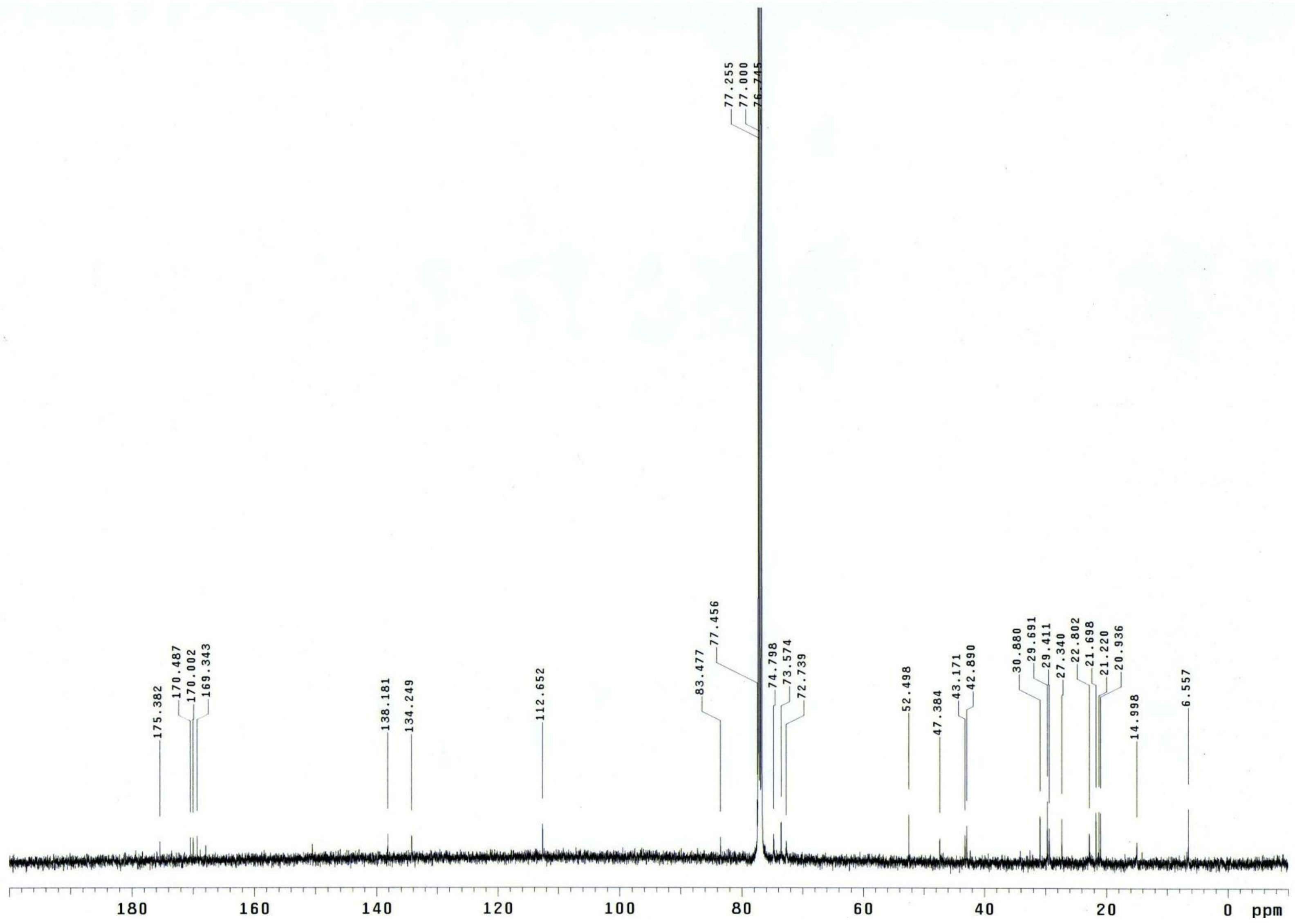

Figure S5. ^{1}H NMR spectrum (400 MHz, $CDCl_3$) of frajunolide R (**3**).

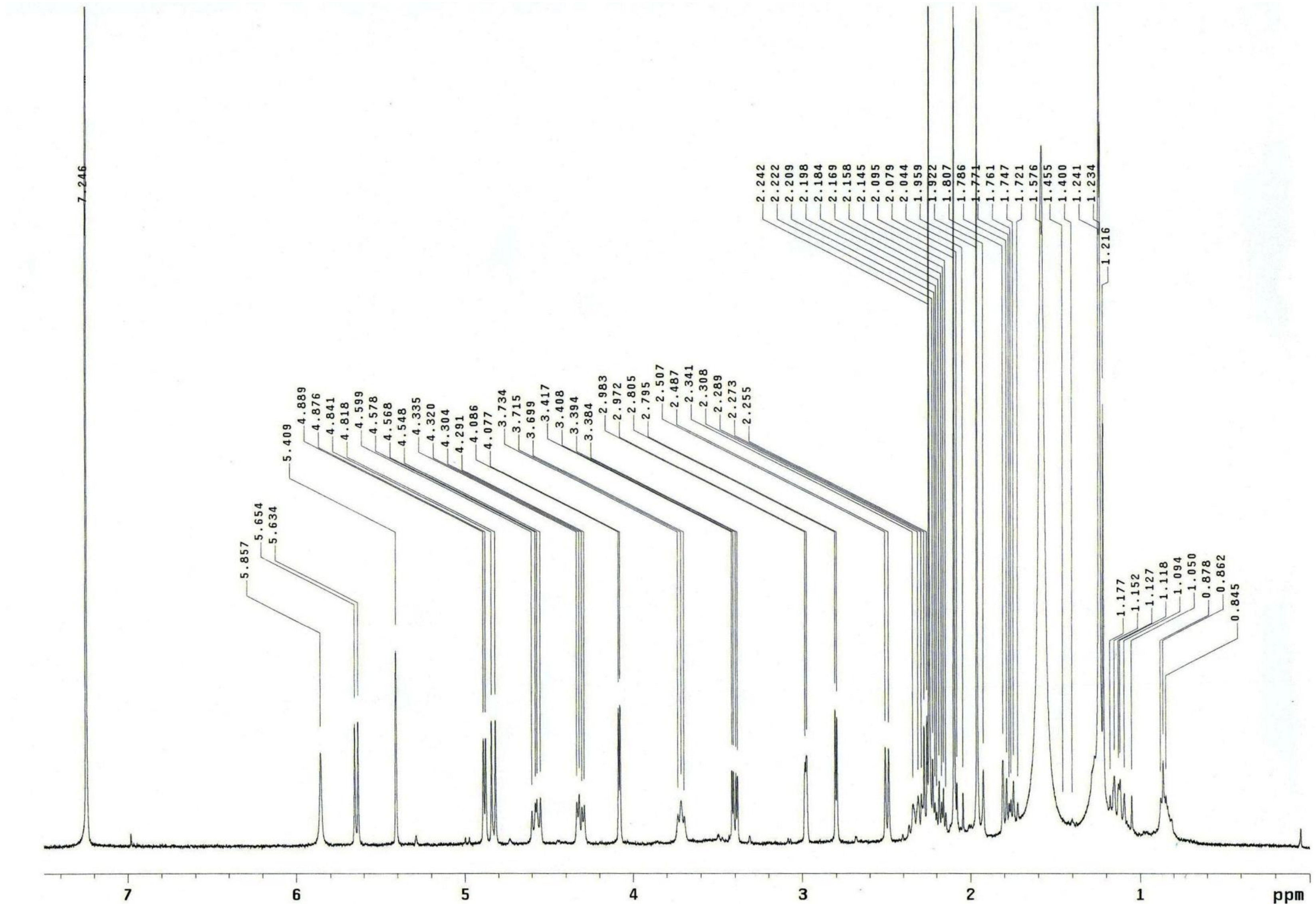

Figure S6. ^{13}C NMR spectrum (100 MHz, $CDCl_3$) of frajunolide R (**3**).

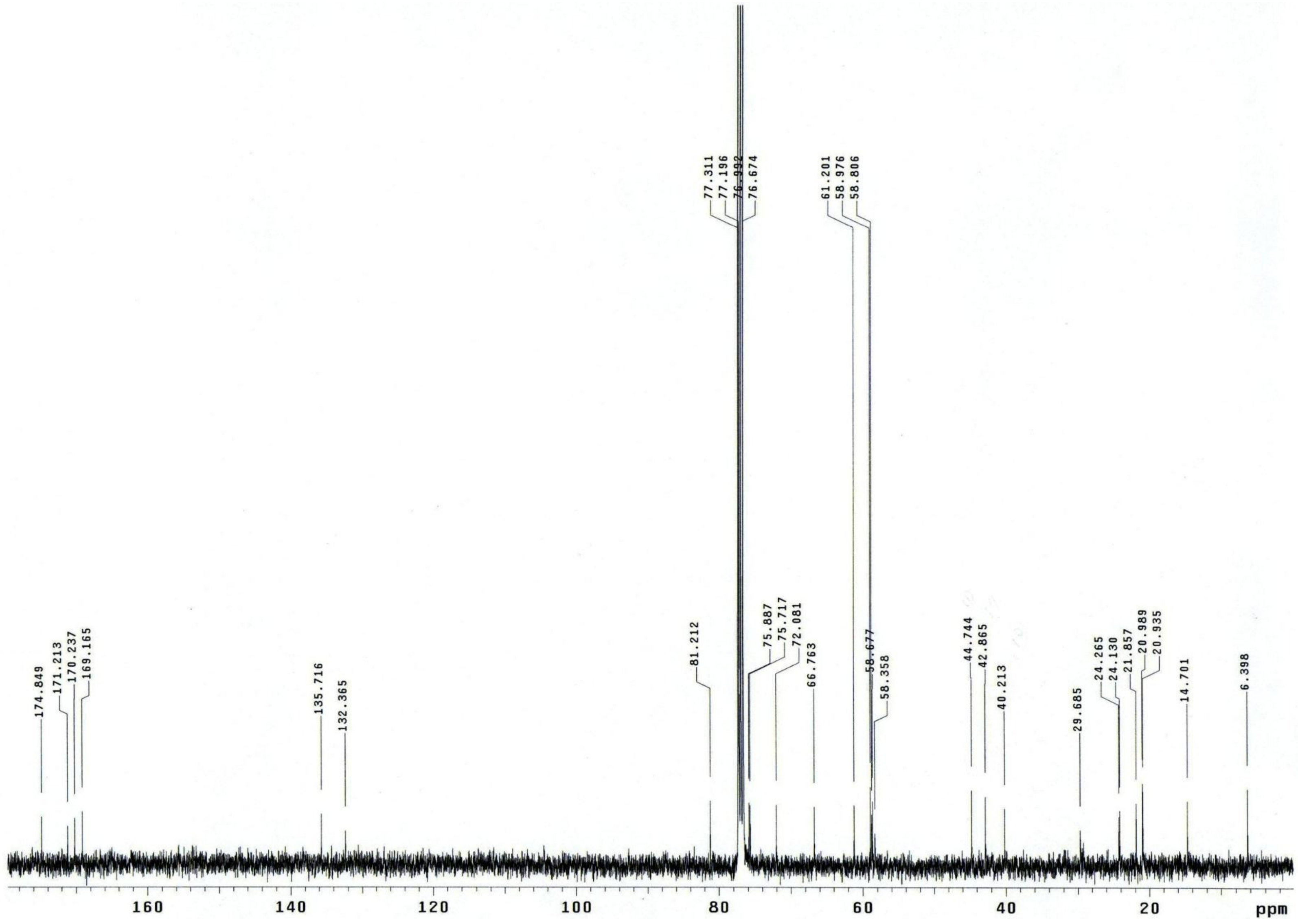

Figure S7. ^{1}H NMR spectrum (400 MHz, $CDCl_3$) of frajunolide S (**4**).

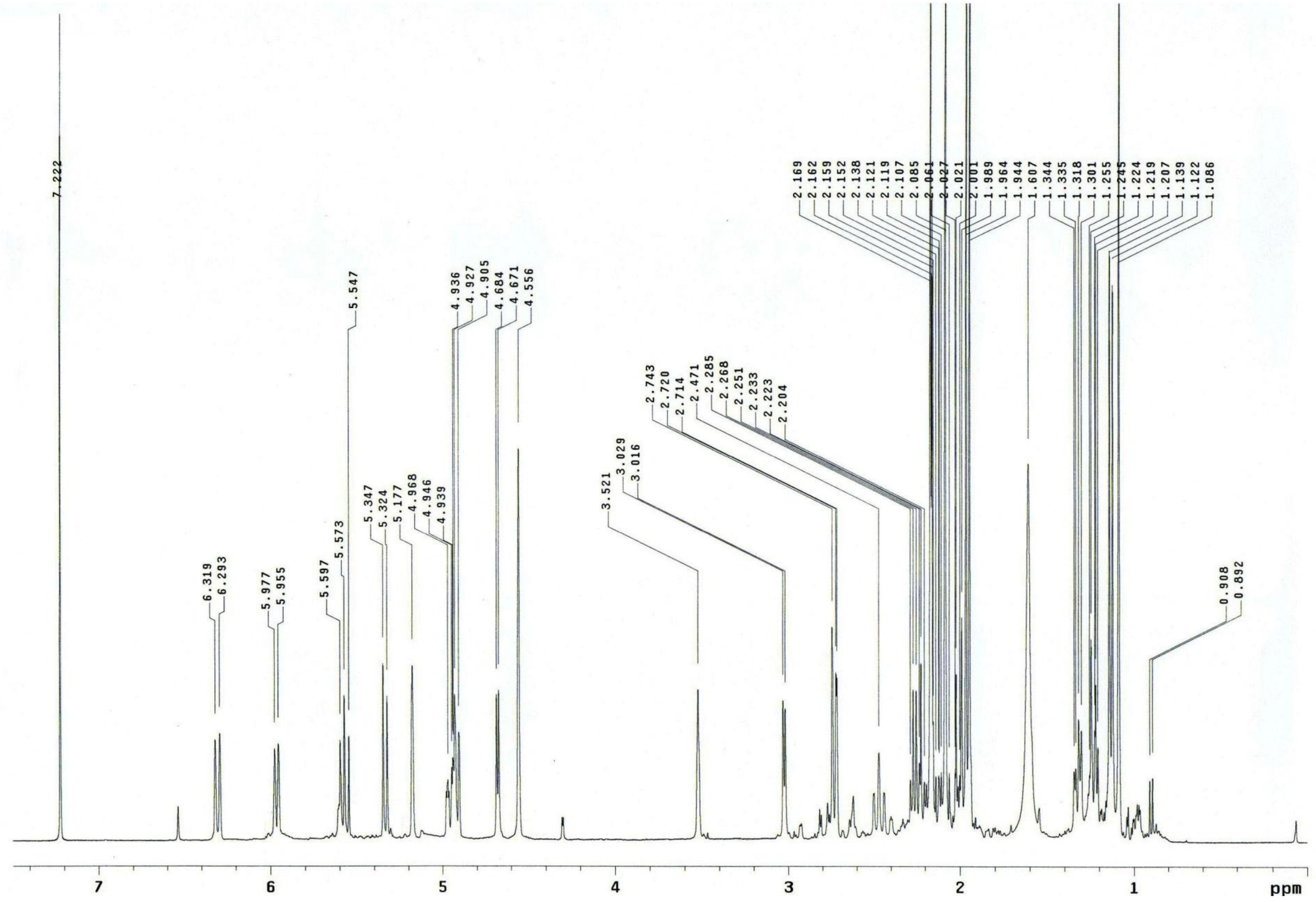

Figure S8. ^{13}C NMR spectrum (100 MHz, $CDCl_3$) of frajunolide S (**4**).

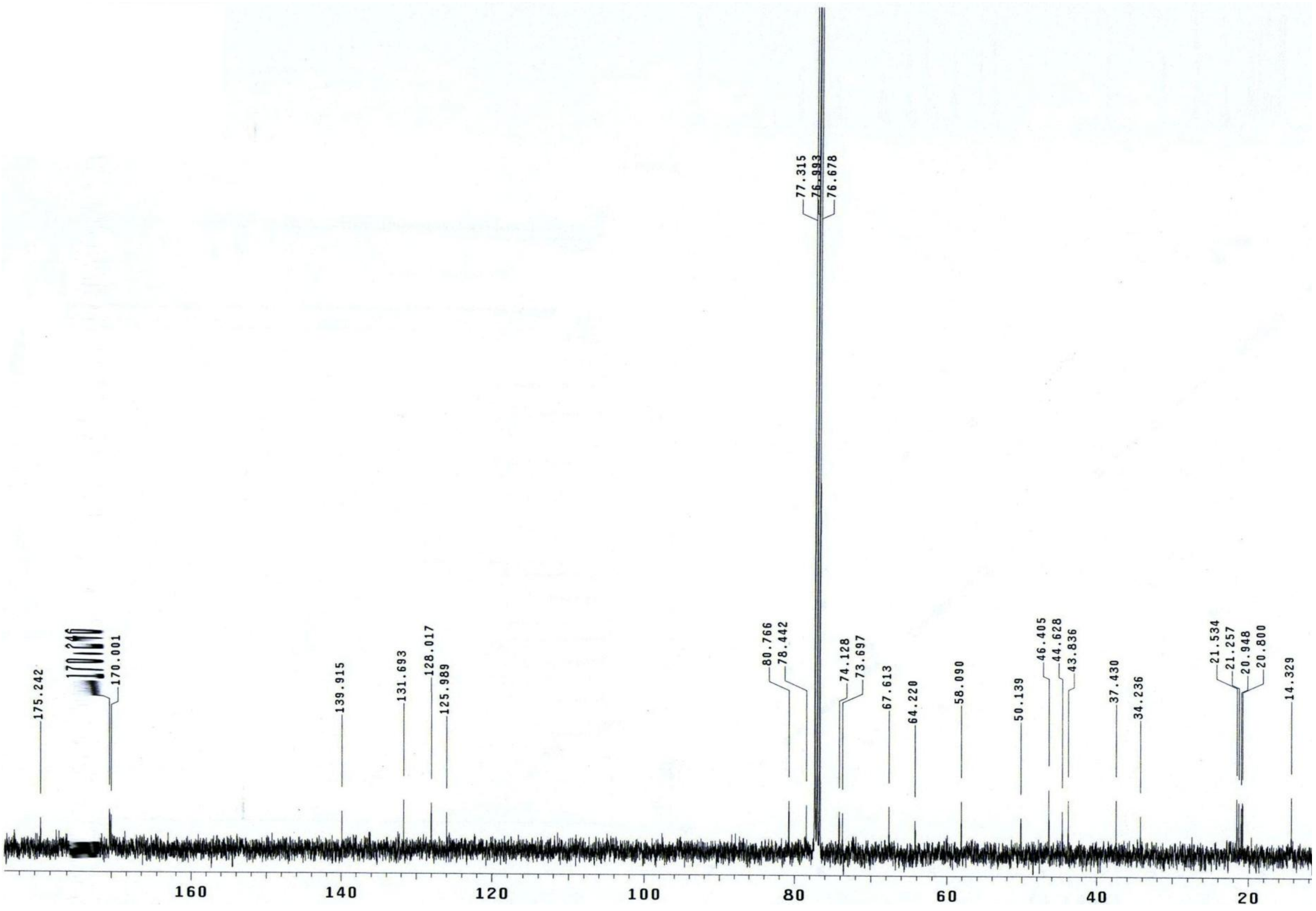

Supplement: Supplementary File 1 — Supplementary Information (PDF, 1096 KB) [file marinedrugs-11-02042-s001.pdf]
